# Fabrication of a PMN-PT Single Crystal-Based Transcranial Doppler Transducer and the Power Regulation of Its Detection System

**DOI:** 10.3390/s141224462

**Published:** 2014-12-19

**Authors:** Qingwen Yue, Dongxu Liu, Wei Wang, Wenning Di, Di Lin, Xi'an Wang, Haosu Luo

**Affiliations:** 1 Key Laboratory of Inorganic Functional Materials and Devices, Shanghai Institute of Ceramics, University of Chinese Academy of Sciences, 215 Chengbei Road, Jiading, Shanghai 201800, China; E-Mails: yue.qingwen@student.sic.ac.cn (Q.Y.); liudxu@outlook.com (D.L.); wwang1008@hotmail.com (W.W.); dwn@mail.sic.ac.cn (W.D.); nick_lindi@163.com (D.L.); wang1972wang@hotmail.com (X.W.); 2 University of Chinese Academy of Sciences, Beijing 100049, China

**Keywords:** ultrasonic transducer, PMN-PT single crystal, transcranial doppler transducer, power regulation

## Abstract

Doppler sonographic measurement of flow velocity in the basal cerebral arteries through the intact skull was developed using a pulsed Doppler technique and 2 MHz emitting frequency. Relaxor-based ferroelectric single crystals Pb(Mg_1/3_Nb_2/3_)O_3_-PbTiO_3_ (PMN-PT) were chosen to be the piezoelectric transducer material due to their ultrahigh piezoelectric coefficients, high electromechanical coupling coefficients and low dielectric loss. The pulse-echo response of the transducer was measured using the conventional pulse-echo method in a water bath at room temperature. The −6 dB bandwidth of the transducer is 68.4% and the sensitivity is −17.4 dB. In order to get a good match between transducer and detection system, different transmission powers have been regulated by changing the impedance of the transmitting electric circuit. In the middle cerebral artery (MCA) measurement photograph results, as the transmission power is increasing, the detection results become clearer and clearer. A comparison at the same transmission power for different transducers shows that the detection photograph obtained by the crystal transducer was clearer than that obtained with a commercial transducer, which should make it easier for doctors to find the cerebral arteries.

## 1. Introduction

Ultrasonic transducers are widely used in medical detection, nondestructive evaluation, underwater sonar and so on [1–3]. The main application of ultrasound in neurology is the examination of extracranial arteries [4,5]. Doppler sonographic measurement of flow velocity in the basal cerebral arteries through the intact skull was developed using a pulsed Doppler technique at 2 MHz emitting frequency [6–8]. In the Transcranial Doppler (TCD) transducer, piezoelectric materials are generally chosen as transduction elements to produce pressure waves at specified frequencies. Traditionally [9,10], lead zirconate titanate (PZT) ceramics are the most popular piezoelectric materials used in the fabrication of ultrasonic transducers because of their high performance and ease of manufacturing [11–14]. Relaxor-PbTiO_3_ (PT) based ferroelectric single crystals near the morphotropic phase boundary (MPB) have been widely researched during the last decades due to their significantly high electromechanical coupling coefficients (*k*_t_ ∼ 60%), extremely large strains (>1%) and extremely high piezoelectric coefficients (*d*_33_ > 2000 pC/N) compared with PZT-based piezoceramics (strain ∼ 0.1%, *k*_t_ ∼ 48%, *d*_33_ ∼ 400–600 pC/N) [15–17].

Because the material properties such as dielectric constant and mechanical quality factor are very different between single crystals and PZT ceramics, when the fabricated transducer is analyzed as an equivalent circuit, the value of the electric impedance in a single crystal-based transducer is bigger than that in a PZT ceramics-based transducer which leads to an impedance mismatch when the single crystal-based transducer works with the current detection systems. In addition, concerning the difference in acoustic impedance between PZT ceramics and PMN-PT single crystals, the acoustic impedance of the matching layer should be carefully concerned when designing the transducer. In this paper, a PMN-PT single crystal-based 2 MHz TCD transducer has been fabricated. Different transmission powers have been regulated by changing the impedance of the transmitting electric circuit to get a better Transcranial Doppler detection effect.

## 2. Preparation of PMN-PT Single Crystals

**Figure 1.**
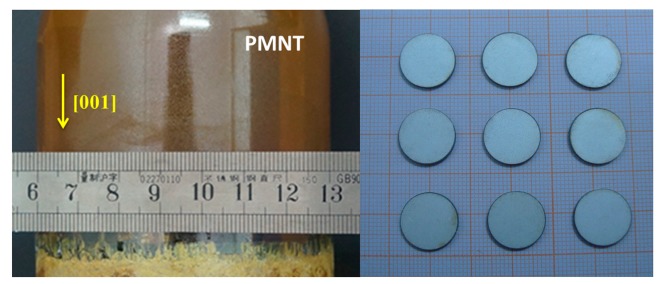
Photograph of the 3-inch PMN-30PT single crystal and ϕ14 mm wafers.

The PMN-0.3PT single crystal compositions were grown directly from their melt by the modified Bridgman technique in SICCAS. The boule size growing along the <001> direction is ϕ76 mm × 80 mm and the available plate size can reach 40 mm × 40 mm. Figure 1 shows a photograph of the 3-inch PMN-30PT single crystal. The direction of the [001] axis in as-grown crystals was determined using an X-ray diffractometer and then the crystal was sliced into (001) wafers. A silver electrode was then printed on the plate and fired at 750 °C for 1.5 h. After the crystals cooled down to room temperature, they were poled under an electric field of 10 kV/cm in silicone oil for 15 min at 90 °C, thus completing the preparation of the crystal resonator.

The electromechanical coupling coefficient *k*_t_ can be calculated using the following equation [18]:

$$k_{t}^{2}=\frac{\pi }{2}\frac{f_{r}}{f_{a}}\tan\left(\frac{\pi }{2}\frac{f_{a}-f_{r}}{f_{a}}\right)$$

where *f*_r_ is the resonant frequency and *f*_a_ is the anti-resonant frequency determined by an HP4294A impedance analyzer, respectively.

## 3. Fabrication and Characterization of the TCD Transducer

### 3.1. Fabrication of the PMN-PT-Based TCD Transducer

PMN-PT wafers (ϕ14 mm) with 2 MHz working frequency were prepared for TCD. In order to enhance the sensitivity and bandwidth, the TCD transducer was designed with air backing and two matching layers. Table 1 shows the basic piezoelectric and acoustic properties of the single crystal and matching layers. The KLM model was used to calculate the ideal acoustic impedances of the first matching layer (*Z*_1_) and second matching layer (*Z*_2_):

$$Z_{1}={\left(Z_{0}^{4}Z_{L}^{3}\right)}^{1/7}$$

$$Z_{2}={\left(Z_{0}Z_{L}^{6}\right)}^{1/7}$$

According to the layer values shown in Table 1, the matching layer was fabricated by using a mixture of different sized tungsten powders and EPO-TEK 301-2 epoxy. The basic acoustic information is shown in Table 2.

**Figure 2.**
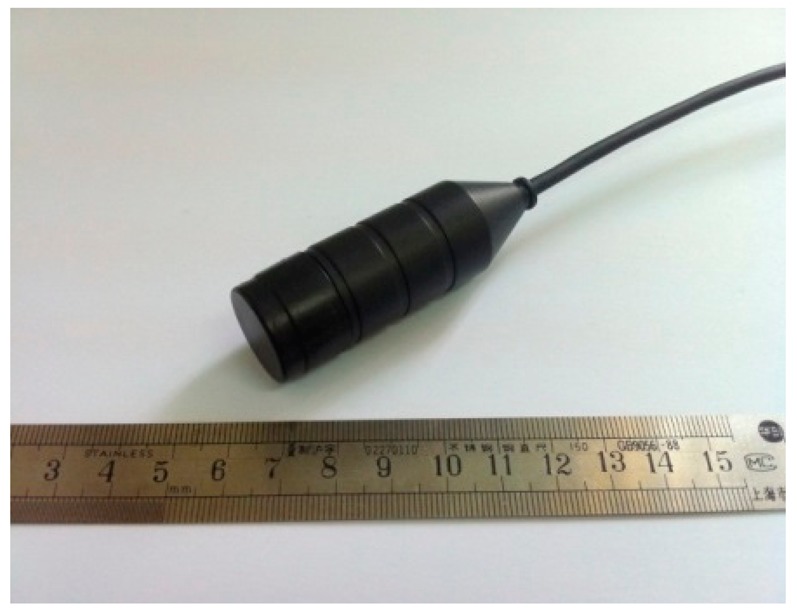
The PMN-PT single crystal-based TCD transducer.

**Figure 3.**
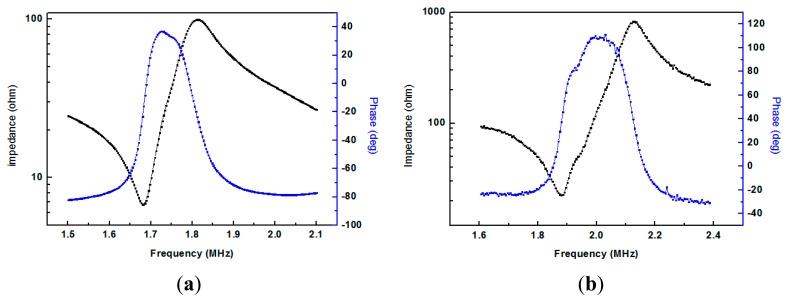
The frequency dependent impedance and phase angle of the as-fabricated TCD transducer: (**a**) PZT ceramic-based transducer; (**b**) PMN-PT single crystal-based transducer.

Figure 2 shows a photograph of the 2 MHz TCD transducer fabricated using a PMN-PT single crystal. The impedance and phase angle spectra of the fabricated TCD transducer measured by a HP4294A impedance analyzer are shown in Figure 3. Compared with the measurement result in Figure 3a,b, the resonant frequency and the anti-resonant frequency are 1.88 MHz and 2.13 MHz, respectively, and the transducer has a maximum phase angle at 2 MHz.

### 3.2. Acoustic Characterization of the TCD Transducer

The pulse-echo response of the transducer was measured using the conventional pulse-echo method in a water bath at room temperature. The transducer was mounted in the water tank in front of a stainless target block which had a plane circular cross-section presented to the transducer. The distance between the transducer and the block was chosen to be the 55 mm, which is the detection distance of the middle cerebral artery (MCA).

**Figure 4.**
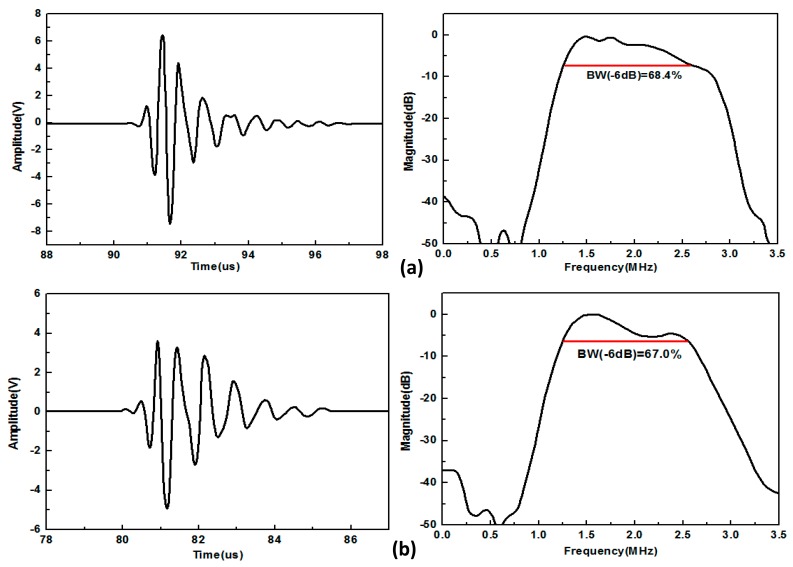
The measured pulse-echo response and frequency spectrum of (**a**) PMN-PT-based TCD transducer and (**b**) commercial TCD transducer.

The transducer was excited by a standard pulser (Model 5077PR, Panametrics, Waltham, MA, USA) with the output voltage of 100 V by employing a variable attenuator. The echo waveforms from the reflection of a stainless steel target were recorded by a digital oscilloscope (Model 54622A, Agilent Technologies Inc. Santa Clara, CA, USA). The frequency spectrum of the transducer echo response was obtained using the fast Fourier transform (FFT) function of the oscilloscope trace. Figure 4 shows the measured pulse-echo waveform and spectrum. The receive voltage is 13.4 V for the PMN-PT single crystal-based transducer and 9.1 V for a commercial transducer which uses PZT-5H ceramics as its active element. The −6 dB bandwidth of the PMN-PT single crystal-based transducer is 68.4%, higher than that of the commercial transducer which is 67.0%. Besides, the two-way sensitivity is −17.4 dB for the PMN-PT single crystal-based transducer and −20.8 dB for the commercial transducer.

## 4. Power Regulation of the Detection System and the Doppler Sonographic Measurement of MCA

### 4.1. The Detecting System

**Figure 5.**
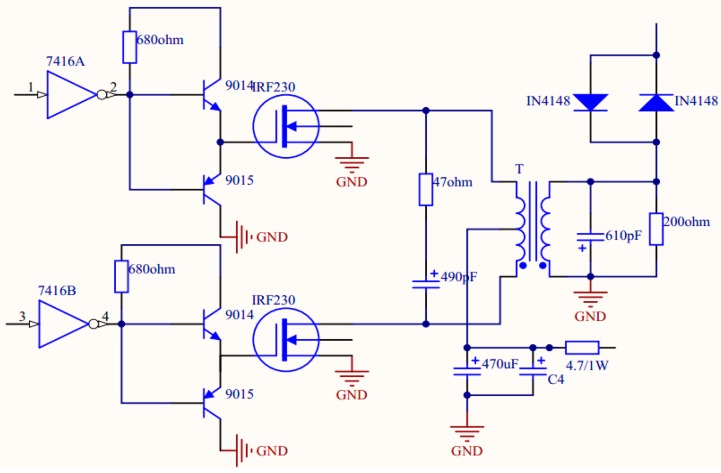
The signal-radiation electric circuit of the detection system.

Table 3 gives the comparison of the transducer impedance and relative dielectric constant between the PMN-PT single crystal-based transducer and a PZT ceramic-based transducer. According to the data in Table 2, the PMN-PT single crystal-based transducer has a much bigger impedance value than the PZT ceramic-based transducer. Figure 5 shows the signal-radiation electric circuit of the detection system. It is a push-pull amplification circuit using two Field Effect Transistors (FETs, IRF230). The two FETs work alternately to amplify the current signal. The coil transformer has two functions, one is to amplify the signal from the FET and the other is to ensure the impedance matching between the electric circuit and transducer. The number of turns in the secondary coil depends on the impedance of the transducer and secondary electric circuit and the turns ratio of primary coil and secondary coil can be calculated by the equation below:

(4) $$N=\sqrt{\frac{Z_{s}}{Z_{p}}}$$

where *Z*_s_ is the impedance of secondary electric circuit and *Z*_p_ is the impedance of primary electric circuit.

In Figure 5, a capacitance and a resistance were used to make a parallel connection with the transducer and secondary coil. The maximum power output for the transducer is under the condition where the imaginary part of the impedance in the secondary electric circuit is close to zero. According to the impedance measurements of the electric circuit and transducer, in order to make the electric circuit have a perfect match for the impedance of the transducer, the turns ratio *N* of the electric circuit for the PMN-PT-based TCD transducer is 4.

### 4.2. Power Regulation and the Doppler Sonographic Measurement of MCA

By using the detection system, the Doppler sonographic measurement of MCA was done under different transmission powers. The transmission power was calculated by the equation below:

(5) $$P=\text{V}I\cos\alpha =\frac{V^{2}}{\left|Z\right|}\cos\alpha$$

where *V* is the voltage of the transducer, *Z* is the impedance of the transducer and α is the phase angle between the voltage and current of the transducer. By changing the impedance of the transmitting electric circuit, four different transmission powers were obtained for the Doppler sonographic measurement of MCA. All the MCA measurements in this experiment were obtained by testing the MCA at the same time under the same measurement conditions. This avoids any measurement differences caused by the liquid information, flow characteristics and skull information and makes it easier to analyse the testing results with the influence of power detection.

**Figure 6.**
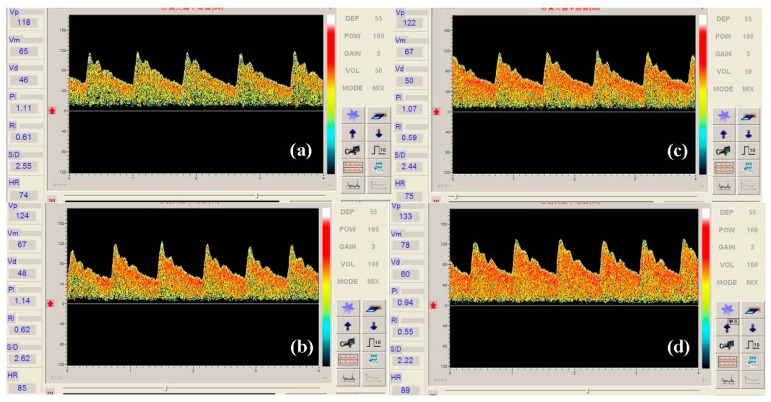
Doppler sonographic measurement of MCA at the transmission powers of (**a**) 2.6 J; (**b**) 11.12 J; (**c**) 15.5 J; and (**d**) 19.8 J.

In the MCA detection photograph, the density of the red pointe is directly proportional to the sensitivity of the transducer. Figure 6 shows the Doppler sonographic measurement of MCA for different transmission powers. From the sonographic measurement we can see that the density of red points in the sonographic measurement of MCA becomes bigger with the transmission power increases, which means the sensitivity of the transducer changes by changing the transmission power.

### 4.3. Comparison of the PMN-PT Single Crystal Based Transducer and Commercial Transducer

As mentioned before, the number of the red dots in the MCA photograph represents the sensitivity of the transducer when detecting the blood vessels. The more the number of red dots obtained in a detection photograph, the higher the sensitivity of the transducer was. However, it is a little difficult to distinguish the detection effect of the MCA measurement using two transducers at full power.

**Figure 7.**
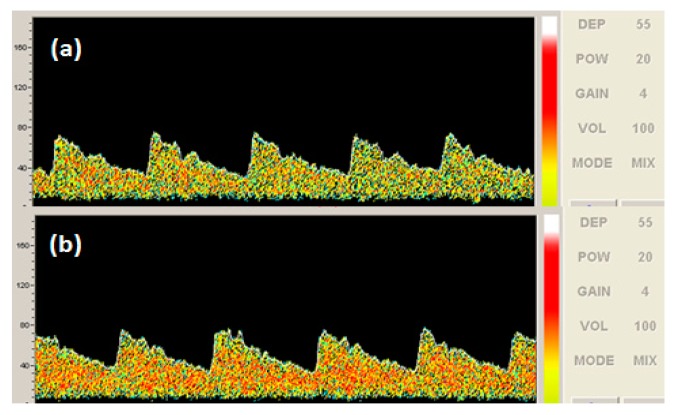
The Doppler sonographic measurement at 20% power: (**a**) commercial transducer; (**b**) PMN-PT single crystal based transducer.

In order to compare the detection effect, 20% power of the detection system was used to obtain the Doppler sonographic measurement of MCA blood flow velocity. The photographic comparison of a commercial transducer and the PMN-PT-based transducer was measured, which is shown in Figure 7. From the photograph of MCA blood flow velocity, the measurement by a commercial transducer has less red area than that measured by the PMN-PT single crystal-based transducer, which means the PMN-PT single crystal-based TCD transducer has a much better detection result than the commercial transducer.

## 5. Conclusions

A relaxor ferroelectric single crystal PMN-PT-based ultrasonic TCD transducer was fabricated and acoustic characterization of the transducer was investigated. The bandwidth and the sensitivity of the TCD transducer have been improved a lot compared with PZT ceramics-based transducers. By changing the impedance of the electric transmitting circuit, different transmission powers were obtained to investigate the Transcranial Doppler detection effect. The results shows a considerable commercial use potential of the PMN-PT single crystal-based transducer with the proper power regulation.

## Figures and Tables

**Table 1.** Piezoelectric and acoustic properties of the single crystal and transducer.

**Layer**	**Properties**	**Values**
PMN-PT single crystal	Density (kg/m^3^)	8000
	Dielectric constant	5500
	*k*_t_	0.62
	Piezoelectric coefficient *d*_33_ (pC/N)	2000
	Sound velocity (m/s)	4600
	Acoustic impedance (MRayl)	36.8

Matching layer 1	Acoustic impedance (MRayl)	9.4

Matching layer 2	Acoustic impedance (MRayl)	2.4

Backing	Acoustic impedance (MRayl)	0.0004

**Table 2.** Properties of the fabricated passive materials used in the TCD transducer.

**Material**	**Use**	**c (m/s)**	**ρ (kg/m^3^)**	**Z (MRayls)**
6.5 μm tungsten powders/EPO-TEK 301-2	Matching layer 1	1430	6520	9.32
EPO-TEK 301-2	Matching layer 2	2350	1120	2.63

**Table 3.** The impedance and relative dielectric constant of the PMN-PT single crystal-based transducer and PZT ceramic-based transducer.

	***f*_r_ (MHz)**	**Impedance @*f*_r_ (ohm)**	***f*_a_ (MHz)**	**Impedance @*f*_a_ (ohm)**	**Impedance @2 MHz (ohm)**	**Dielectric Constant**
PZT ceramic-transducer	1.68	6.65	1.81	99.5	37.57	3218.7
PMN-PT SC-transducer	1.88	22.36	2.13	825.7	589.8	4629.7

## References

[1] Delcker A. (1996). Applications of ultrasound in neurological diagnostics. Med. Welt.

[2] Hamann H., Vollmar J.F. (1986). Carotid endarterectomy in patients with vertebrobasilar insufficiency. Langenbecks Arch. Fur Chir..

[3] Huang C.C., Chen R.M., Tsui P.H., Zhou Q.F., Humayun M.S., Shung K.K. (2009). Measurements of attenuation coefficient for evaluating the hardness of a cataract lens by a high-frequency ultrasonic needle transducer. Phys. Med. Biol..

[4] Li X., Luo H. (2010). The Growth and Properties of Relaxor-Based Ferroelectric Single Crystals. J. Am. Ceram. Soc..

[5] Luo H.S., Xu G.S., Wang P.C., Yin Z.W. (1999). Growth and characterization of relaxor ferroelectric PMNT single crystals. Ferroelectrics.

[6] Matsuoka N., Paeng D.G., Chen R.M., Ameri H., Abdallah W., Zhou Q.F., Fawzi A., Shung K.K., Humayun M. (2010). Ultrasonic Doppler measurements of blood flow velocity of rabbit retinal vessels using a 45-MHz needle transducer. Graefes Arch. Clin. Exp. Ophthalmol..

[7] Shung K.K., Cannata J.M., Zhou Q.F. (2007). Piezoelectric materials for high frequency medical imaging applications: A review. J. Electroceram..

[8] Wanga H.X., Luob H.S. (2005). Dependence of dielectric properties of 0.9Pb(Mg_1/3_Nb_2/3_)O_3_-0.1PbTiO_3_ crystals on the crystallographic orientation and external electric field. Ferroelectrics.

[9] Sun E., Cao W. (2014). Relaxor-based ferroelectric single crystals: Growth, domain engineering, characterization and applications. Prog. Mater. Sci..

[10] Sun P., Wang G.F., Wu D.W., Zhu B.P., Hu C.H., Liu C.G., Djuth F.T., Zhou Q.F., Shung K.K. (2010). High Frequency PMN-PT 1-3 Composite Transducer for Ultrasonic Imaging Application. Ferroelectrics.

[11] Totaro R., Corridoni C., Marini C., Marsili R., Prencipe M. (1993). Transcranial Doppler evaluation of cerebral blood-flow in patients with paroxysmal atrial-fibrillation. Ital. J. Neurol. Sci..

[12] Totaro R., Marini C., Cannarsa C., Prencipe M. (1992). Reproducibility of transcranial Doppler sonography: A validation-study. Ultrasound Med. Biol..

[13] Volc D., Possnigg G., Grisold W., Neuhold A. (1988). Transcranial dopplersonography of the vertebro-basilar system. Acta Neurochir..

[14] Wang W., Li X.B., Or S.W., Leung C.M., Jiao J., Zhang Y.Y., Zhao X.Y., Luo H.S. (2012). Temperature dependence of dielectric polarization and strain behaviors for rhombohedral PIMNT single crystal with different crystallographic orientations. J. Alloys Compd..

[15] Wang W., Or S.W., Yue Q.W., Zhang Y.Y., Jiao J., Ren B., Lin D., Leung C.M., Zhao X.Y., Luo H.S. (2013). Cylindrically shaped ultrasonic linear array fabricated using PIMNT/epoxy 1-3 piezoelectric composite. Sens. Actuators A Phys..

[16] Xu G.S., Luo H.S., Guo Y.P., Xu H.Q., Qi Z.Y., Zhong W.Z., Yin Z.W. (2001). Growth and piezoelectric properties of Pb(Mg_1/3_Nb_2/3_)O_3_-PbTiO_3_ crystals by the modified Bridgman technique. Solid State Commun..

[17] Yu P., Wang F., Zhou D., Ge W., Zhao X., Luo H., Sun J., Meng X., Chu J. (2008). Growth and pyroelectric properties of high Curie temperature relaxor-based ferroelectric Pb(In_1/2_Nb_1/2_)O_3_-Pb(Mg_1/3_Nb_2/3_)O_3_-PbTiO_3_ ternary single crystal. Appl. Phys. Lett..

[18] Pijolat M., Loubriat S., Queste S., Mercier D., Reinhardt A., Defay E., Deguet C., Clavelier L., Moriceau H., Aid M. (2009). Large electromechanical coupling factor film bulk acoustic resonator with X-cut LiNbO_3_ layer transfer. Appl. Phys. Lett..

